# Translation and cross-cultural adaptation of the Integrated Palliative Care Outcome Scale in Hindi: Toward capturing palliative needs and concerns in Hindi speaking patients

**DOI:** 10.1177/02692163221147076

**Published:** 2023-01-31

**Authors:** Tushti Bhardwaj, Rachel L Chambers, Harry Watson, Srividya, Irene J Higginson, Mevhibe B Hocaoglu

**Affiliations:** 1 Cicely Saunders Institute of Palliative Care, Policy and Rehabilitation, King’s College London, UK; 2 Dr. Bhim Rao Ambedkar College, University of Delhi, Delhi, India; 3 LifeVidya, Delhi, India; 4 King’s College Hospital NHS Foundation Trust, Denmark Hill, UK

**Keywords:** Patient reported outcome measures, palliative care, end of life care, cross-cultural comparison, cancer patients, India, focus groups

## Abstract

**Background:**

Culturally relevant patient-centered outcomes tools are needed to identify the needs of patients and to assess their palliative care concerns.

**Aim:**

To translate and culturally adapt the Integrated Palliative Care Outcome Scale (IPOS) into Hindi.

**Design:**

The study applied a standardized methodology entailing six phases for translation and content validation: equivalence setting through a three-step process; forward translation; blind backward translation; expert review by a panel of the POS team; cognitive de-briefing with patients; and proof-reading of the final tool. All interviews and focus groups were audio-recorded, transcribed and analyzed using content analysis.

**Setting/participants:**

**(**1) Healthcare professionals including doctors, nurses, psychologists, counselors, and volunteers working in Indian palliative care settings with expertise in both English and Hindi languages; (2) Hindi speaking patients diagnosed with cancer who were receiving palliative care in community settings. Caregivers, palliative care experts, and language translators contributed to the translation procedure.

**Results:**

Phrases like nausea, poor appetite, drowsiness, and depression were difficult to translate into Hindi. Response categories “occasional” and “sometimes” were overlapping. All items, instructions and response categories were simple to understand. A visual thermometer is a unique feature of Hindi IPOS to facilitate responses from less educated patients.

**Conclusion:**

Hindi IPOS has face and content validity for use in clinical practice and research. The Hindi IPOS has implications beyond Indian palliative care settings. Millions of Hindi speakers can now respond to IPOS, and have a tool for communicating their palliative care needs in their mother tongue to inform patient-centered care.

**What is already known about the topic?**

- Culturally relevant and valid psychometric patient-centered outcome tools to assess palliative care concerns are critical for channeling resources where they are needed most
- Cultural and local contexts must be considered when using patient-centered outcomes in different settings
- Integrated Palliative care Outcome Scale (IPOS) is a clinically relevant, reliable, valid, brief patient-centered outcome tool for capturing the multidimensional impact of symptoms and concerns in advanced illness

**What this paper adds?**

- This paper highlights the importance of cultural adaptation, moving beyond translation to exploring, rephrasing and communicating meanings and concepts clearly in diverse cultures
- This study provides insight into personalized assessments of concerns and needs within a specific population, allowing for a more holistic approach to person-centered care for Hindi speaking patients
- This paper demonstrates the importance of cognitive interviews and focus group discussions in ensuring content validity

**Implications for practice**

- Integration of the Hindi IPOS for use in care settings will support person-centered and a holistic approach to care that meets individual needs
- Hindi IPOS could be used to identify patient’s unmet needs and make the most of resources to address them
- Features of Hindi IPOS such as the thermometer scale support feedback from patients who may have different educational backgrounds

## Introduction

Since the introduction of palliative care in India in the 1980s the need for palliative care has continued to grow.^1–3^ In low to middle-income countries such as India, 78% of those who require palliative care are not receiving it.^4^ A person-centered outcome tool will support palliative care services, focusing resources where it is needed most.^5^ Palliative care practice requires a personalized assessment of patients’ symptoms, priorities, needs, and concerns for the best delivery of person-centered care.^4^ To facilitate this, culturally relevant, brief, valid person-centered outcome tools are needed to evaluate palliative care services and the unmet needs of patients. In Indian settings, there are condition specific patient centered outcome measures (PCOM) available in Hindi that are used in clinical research and practice^6,7^. However, a valid and brief PCOM focusing on life-limiting and advanced illness is relevant and beneficial to wider groups of patients. The Palliative Care Outcome Scale (POS) family of measures are person-centered outcome tools for patients living with life-limiting illnesses.^8,9^ POS measures have been validated in persons living with cancer, respiratory, heart, renal, liver failure, and neurological disorders.^10–16^ The Integrated Palliative Outcome Scale (IPOS) belongs to the POS family of measures and has been translated into more than 16 languages, and is widely used with patients receiving palliative care services, demonstrating its usefulness to clinical practice and research. IPOS is a brief 10-item scale that has questions about physical symptoms, mood, family anxieties, practical concerns and financial needs. IPOS is a brief person-centered outcome tool that supports the integration of palliative care in resource limited settings, where prioritization of the most urgent patient needs is required. Cross-cultural adaptation of psychometric instruments has been recommended by researchers to account for the ‘cultural context and lifestyle of the target population’,^17,18^ and to limit the risk of introducing bias into the study and producing misleading results. This study aimed to translate and culturally adapt IPOS in Hindi and to ensure the measure is conceptually equivalent, relevant, and compatible with cultural patterns, meaning, and values for Hindi speaking populations.

## Methods

### Design

**Figure 1.**
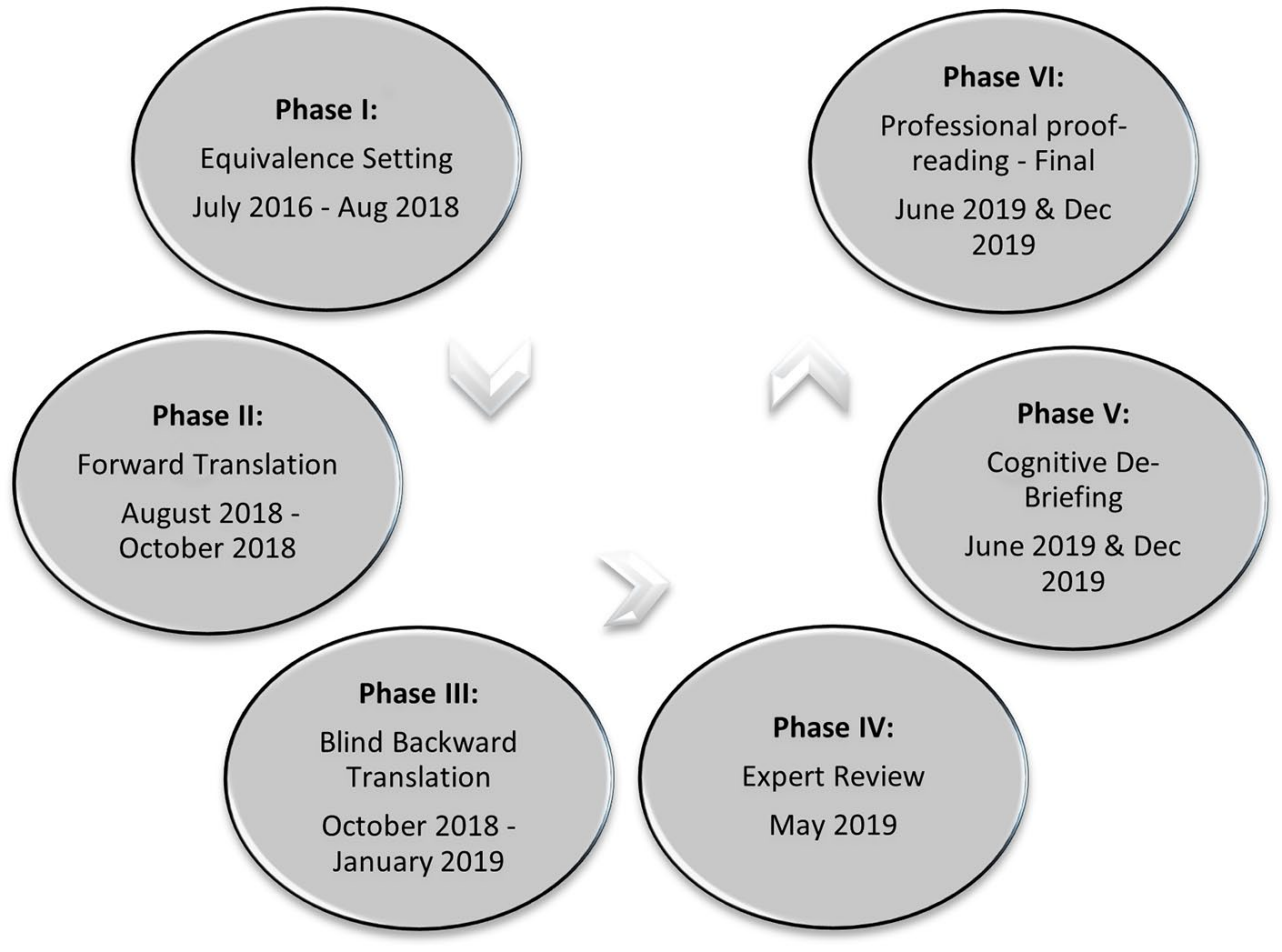
Translation and cross-cultural adaptation process.

A translation and cultural adaptation study was carried out in six phases summarized in Figure 1 and detailed subsequently. Standard methodologies prescribed by the POS development team^19^ and guidelines published by the European Organization for Research and Treatment of Cancer (EORTC) were used.^20^

Six phases were followed to translate and culturally adapt the instructions, individual items, and response options (Figure 1). For the first phase, equivalence setting, we conducted a scoping literature search, cognitive testing with a purposive sample of seven palliative care professionals and two focus group discussions with patients. For the second phase, forward translation in Hindi was completed by three independent translators. For the third phase, blind backward translation was completed by three additional independent translators. For the fourth phase, the backward translation version was reviewed by the POS expert panel. During the fifth phase, qualitative pretesting and de-briefing were conducted with Hindi speaking patients through in-depth interviews. Data collection began in July 2017 and ended in December 2019 (TB & S).

### Phase I: Equivalence setting

#### Scoping literature search

We conducted a scoping literature search to identify Patient Reported Outcome Measures used in Indian settings and to identify the most common concerns reported by Hindi speaking patients receiving palliative care services. We searched MEDLINE (1966–2018) and updated the search in 2021 (1966–2021) through PubMed and the Indian Journal of Palliative Care. We used the following keywords “palliative care” and “end of life care”.

#### Face to face interviews with professionals

We conducted semi-structured interviews with professionals using a topic guide (see Supplementary Appendix 1). Questions focused on comprehension (“What does this question from IPOS English mean to you in your own words?”), retrieval (“-How well could you remember your experience when answering this question?”), and response patterns of each item of the IPOS. Conversations took place in English. All interviews were audio-recorded, transcribed, and analyzed using content analysis by TB & S.

#### Face to face focus group discussions with patients

Two focus group discussions were conducted in CanSupport clinics to understand if the concepts in IPOS were recognized by Hindi speaking cancer patients. Each group consisted of eight to nine adults with different cancer diagnoses and lasted approximately one hour. A topic guide (which was drafted based on responses from face-to-face interviews with professionals in the prior step) was used to explore cognitive processes for each item (see Supplemental Appendix 2). At this stage, interpretation of words was explored. Focus group discussions were audio-recorded, and a researcher made field notes. Two researchers with experience in qualitative research (TB & S) listened to the audio recordings and prepared a report. The report captured whether the concepts used in IPOS were relevant to the culture before moving on to the next phase.

### Phase II: Forward translation

The IPOS was translated into Hindi with the help of three translators: 1) a palliative care expert, 2) a language expert, and 3) a family caregiver. All were native Hindi speakers, fluent in English. The first two translators produced two different and independent translations of IPOS. These two versions were reviewed by a an independent family caregiver and the lead author of this study (TB). A detailed report of the process of translation, challenging phrases in the Hindi language, and mutually agreed upon decisions were prepared and presented to for the POS development team for approval ahead of Phase III.

### Phase III: Blind backward translation

Three translators were recruited, all were blind to the original IPOS. The first two translators, a palliative care doctor and a language expert independently translated the Hindi IPOS back to English, these were then compared to the original IPOS by a third translator, a family caregiver to ensure consistency. Any inconsistencies between the Hindi and original English version were addressed by the POS development team in Phase IV.

### Phase IV: Expert review

A member of the POS development team at the Cicely Saunders Institute, King’s College London, together with the lead author of this paper, reviewed and discussed the pre-final version and the methods used. MH and a native Hindi speaking independent reviewer reviewed the Hindi IPOS and suggested changes. The pre-final Hindi version was drafted and agreed upon before progressing to the next phase.

### Phase V: Cognitive de-briefing

A second sample of patients attending palliative care outpatient clinic appointments at CanSupport were approached to pilot whether instructions, items, and response categories of Hindi IPOS were clear. Patients read each item and were asked to think aloud, providing their feedback and suggestions on how items, instructions, or responses could be improved. Patient feedback was reported to the POS development team for review. MH and TB discussed the suggestions which were incorporated into the final version of Hindi IPOS.

### Phase VI: Professional proofreading

The final versions of the Hindi IPOS for staff and patients in hospital and community settings were proofread by a professional Hindi language editor. The Hindi IPOS was published on the POS website.

### Setting

This research was implemented at CanSupport—a charity that offers home palliative care services in Delhi, national capital regions including Ghaziabad, Noida, Gurgaon, Merrut, and one of the neighboring states, Punjab—through multidisciplinary community teams.

### Ethical approval

The study was approved by the CanSupport Research Ethics Committee.

### Population

From Phase I to Phase VI the following individuals were invited to support the translation and cross-cultural adaptation process: who met the following inclusion criteria:

#### Inclusion criteria

##### Patients:

Hindi speaking patients over the age of 18 attending the outpatient palliative care clinic provided by CanSupport between June 2019 and December 2019. Patients needed to have capacity to consent. Patients who were unable to communicate or too unwell to participate were excluded.

##### Professionals:

Professionals who provided palliative care services at CanSupport and a tertiary care hospital.

Key bilingual academics, family caregivers and professional editors were identified by TB and invited to participate in forward and backward translations of Hindi IPOS.

## Results

Table 1 summarises the characteristics of participants who took part in this study.

**Table 1.** Characteristics of patients and translators across various stages.

Participants	Gender	Age*
Patients involved in focus group discussion (Phase I)		
Eight patients (Focus group discussion I)	4F	47*
Nine patients (Focus group discussion II)	3F	44*
Professionals involved in forward translation (Phase II)		
Hindi language editor	M	65
Medical doctor	F	58
Caregiver	F	35
Professionals involved in backward translation (Phase III)		
English language expert	F	53
Medical doctor	M	40
Cancer survivor	F	42
Patients involved in cognitive de-briefing (Phase V)		
Six patients	3F	39*
Professionals involved in proofreading (Phase VI)		
Professional Hindi language editor	M	65

* Median age reported.

### Phase I: Equivalence setting

The scoping search for identifying PROMs used in Indian settings returned 9 relevant articles from PubMed and 100 articles from the Indian Journal of Palliative Care. We found that few scales are available in Hindi, namely the European Organization for the Research and Treatment of Cancer Quality of Life Questionnaire (EORTCQLQ)^30^ generic along with disease-specific measures,^6,7^ World Health Organization Quality of Life Questionnaire (WHOQLQ 100 & WHOQLQ-BREF), EQ5D/3D,^21–23^ General Health Questionnaire GHQ,^24^ Patient Health Questionnaire PHQ^25^. These tools are widely used to understand the quality of life of cancer patients in an Indian context, but culturally valid palliative care specific instruments are lacking.

The most common concerns reported by Hindi speaking patients receiving palliative care services were pain, dyspnea, restlessness, bowel obstruction, depression, anxiety, stress, poor quality of life, changes in sleep, appetite, energy, spiritual/religious concerns, family emotional issues, social security, and financial needs.^26–41^ A brief review of available tools and literature suggested that concerns raised in the IPOS were holistic and covered all concepts confirming face and content validity.

Table 2 represents the characteristics of seven professionals who participated in IPOS Hindi cognitive testing. All patients were receiving community palliative care services. One group had equal representation of gender while another had 2:1 for male and female respectively.

**Table 2.** Characteristics of professional participants from cognitive testing interviews.

Job title (No. of professionals)	Setting	Age* (at data collection)	Experience range (years)	Education
Clinical team (Nurses and doctors)^4^	Hospital & Home Care	42	1–19	Degree in Nursing, Medicine with palliative care specialization
Other Support professionals and volunteers^3^	Hospital & Home care	57	4–17	M.A (Psychology) and M.Phil. Community health, Law

* Median age is reported.

Professionals and patients did not agree with the use of the following terminology: “concerns,” “anxious”, “depression”, “peace”, “practical problems”. The professionals suggested replacing the word “concerns” with “problems,” as they felt that the Hindi definition of the word “problems” gave better clarity. It was agreed that the word “anxious” would be replaced with “tension”, “peace” with “peace at heart”, and “depression” would be supplemented by explanatory phrases such as “feeling sad or low” to avoid any misunderstanding with clinical depression. The phrase “practical problems” in the last item of the original IPOS was changed to “illness and treatment related” to make it more specific. Explanatory phrases like “personal problems” and “financial problems” were retained for clarity.

The two response categories namely “occasional” and “sometimes” were often overlapping. Professionals felt the phrase “overwhelmingly” indicated a degree of emotion rather than physical symptoms. Table 3 represents the IPOS phrases which were difficult to understand along with their most appropriate equivalent Hindi phrase as suggested by professionals.

**Table 3.** Challenges with some phrases used in the original IPOS in adapting them to the Indian setting and their equivalent phrase/term.

Original IPOS phrase	Equivalent Hindi phrase	Comments by professionals in palliative care
Concern	Problem (परेशानी)	*The question is easy to understand but I think we can remove the word “Concern” only “Problems” is sufficient. (ACH05) I would umm. . .I would say* *“Yes, what are your main problems?” (ACH05)* *That’s what I would like [rather] than concerns. Concerns would be a little bit difficult to answer. (CNS07)*
Nausea	Feelings of vomiting (उल्टी का अहसास / उल्टी जैसा लगना)	*Nausea.. needs to be changed. We can.. ask them if you are feeling sick. (ACH05) Nausea. . .No, you will have to say vomiting. Nausea is the feeling. . .they won’t understand. A patient will only relate to vomiting. Nausea as a category they will not understand. May be if we say . . ..“feelings of vomiting” (CNS07)*
Vomiting (being sick)	Vomiting (उल्टी आना)	*This item is ok once translated to the local language and being sick needs to be dropped as it means not being well in our context (CNS06)*
Anxious	Tense/Worried (तनावग्रस्त/चिंतित)	*[I] want to drop the word. . ..anxious and want to retain the word “Being worried”. [The] patient may not be very clear about the word anxious(CNS03). We can put “Have you been feeling tense?” because with “anxious” [a patient may] get confused.(ACH05)*
Constipation	Inability to pass stool (कब्ज)	*“Constipation” . . . this word is difficult for the patient. Some patients may call hard stool as constipation instead of inability to pass stool for a particular duration let’s say 48–72 hours. Instead of constipation you would like to replace it with “hard stool” or “inability for the bowel movement” (CNS03)*
Depression	Feeling sad, hopeless, lonely, crying often (उदास, निराश, अकेला महसूस करना, बार-बार रोना)	*Sometimes patient also take depression as a sickness. . . separate sickness. . .. . .. So, they may not be comfortable using this term. (CNS03* *Instead of “depression,” we use the word sad, “Sad ” is a common word, for depression they may think, they are patient or mentally ill. . .(ACH05) Depressed many times is a strong word, because depressed is basically a psychiatric or psychological term which is clinical, So, sadness instead of depression (CNS06)*
Feeling peace	Having peace at heart (दिल में शांति हो/ मन शांत होना	*Somebody who is like suffering from cancer. . .. would never get at peace. (CNS01)* *for the last 10 years I am working with patients and . . .. eh. . . they might not attain that peak of peace. . .. because they are always worried. Because there is cancer. . .. (CNS04) Not clear (ACH02) This is a difficult question, they won’t understand. . . (CNS07)*
As much as you wanted	Sufficient (पर्याप्त)	*This is a bit confusing for me too. . … What does this mean? We have to elaborate. . . information about what? We have to give them [a] very specific thing to answer. (CNS03)* *What I think is . . . question is little bit lengthy. (ACH05) We may say : do you have sufficient information, about the illness or treatment(CNS04)*
Practical problems	Personal and financial problems (व्यक्तिगत और वित्तीय समस्याएं)	*Practical problems???? they won’t know what is practical. . .. . .and what is impractical?(CNS03)* *Any practical problem. . .. . . so is that like after the treatment (CNS04) they may not understand it if we look at it from patient’s point of view. (CNS01)*
Occasional	Very rare (बहुत दुर्लभ/ बहुत कम)	*occasionally and sometimes they may confuse. For sometimes they may think its occasionally. Use “Rare” instead of occasionally, (CNS03) *occasionally is not a good, (CNS07)**
Overwhelmingly	Most of the time (अधिकांश समय/ अधिकत्तर समय)	*Overwhelming is an emotional type [of] thing . . ..(ACH05)* Patients [will] understand it better if you [do not put] overwhelmingly. . .″ (CNS04)

### Phase II: Forward translation

**Figure 2.**
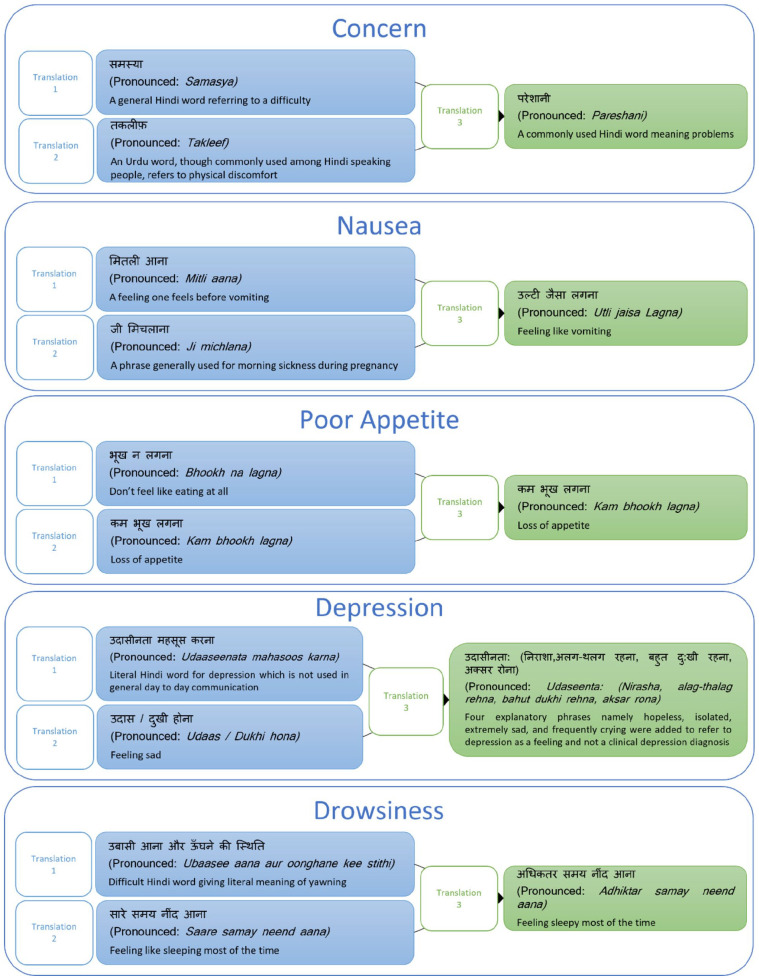
The process of translation and reaching a consensus for difficult phrases in Hindi. Translation 1: translation given by translator 1, Translation 2: translation given by translator 2, Translation 3: consensus between translator 3 and the research team.

During forward translations, some phrases were translated differently by the two translators. The research team and the third translator discussed the variations until a consensus was reached. The Hindi meanings of nausea such as “*mithli aana”* or “*ji michlana”* are not commonly used in day-to-day language or are often used in the context of pregnancy. Therefore, the research team in consultation with the third translator chose a more common phrase “*ulti jesa lagna”* (feeling like vomiting). “Poor appetite” when translated in Hindi means “loss of appetite,” so a commonly used word “*kam bhoog lagna”* was chosen. For drowsiness “feeling sleepy most of the time” was chosen after a lengthy discussion with all translators. The literal Hindi meaning of depression was not commonly used in day-to-day language so explanatory phrases were added to convey that depression did not mean a clinical diagnosis. Explanatory phrases included feeling hopeless, isolated, extremely sad, and frequently crying. Item eight on knowledge and item tenth on practical problems of the original IPOS were difficult to translate. After discussion, the research team finalized a phrase that meant “sufficient information about disease and treatment” rather than “complete knowledge of illness.” For the item on practical problems, it was agreed that the Hindi word meaning “solutions to personal problems were given to the patient” would be used. Figure 2 represents the process of translation and reaching a consensus for difficult phrases in Hindi.

### Phase III: Blind backward translation

Two independent translations were similar in meaning but the sentence structure and grammar differed. In one translation, sentence structure was in dialog format whilst the other was in instruction format. The third translator and the research team unanimously decided to choose the dialog format as it was closer to the original IPOS and extended a personal touch for patients.

The backward translations of the difficult phrases: “nausea”, “drowsiness”, “poor appetite” had similar meaning to the items used in the original IPOS suggesting appropriate use of Hindi words for these phrases.

### Phase IV: Expert review

Following detailed discussions, numeric order was added to the response categories along with Hindi words. MH suggested the use and testing of a visual thermometer (Visual Analog Scale) together with a pictorial scale adopted from the African Palliative Care Association African Palliative Outcome Scale (African APCA African POS)^42^ to facilitate responses from persons who were not able to read and write.

### Phase V: Cognitive de-briefing

The cognitive de-briefing of the pre-final Hindi IPOS version with six palliative care patients suggested that all items, instructions, and response categories were clear and understandable. Patients enjoyed responding using their fingers, but two patients were confused while answering reverse scale items where higher numbers indicated better status. Patients found it easier to indicate their response using the thermometer.

## Discussion

### Validity of IPOS within a Hindi setting

The cross-culturally adapted IPOS Hindi is freely available for use in Indian palliative care settings from the POS website (https://www.pos-pal.org). The Hindi IPOS is conceptually equivalent to the original English version. Several concepts proved challenging to translate and adapt to Hindi including. words such as “concerns”, “problems”, “nausea”, “depression” and “anxious”. These words have also proved difficult to adapt in French^43^ and German^44^ IPOS. Another challenging concept was “being at peace”. This concept was also found to be difficult to translate to French, but was found to be more familiar in Turkish^45^ settings. We took extra care to deeply explore these items with professionals during cognitive interviewing. Professionals state that diseases like cancer bring challenges to the patient, not only for their own physical and emotional health, but for their family life, related responsibilities, social connections, and their work-life balance. Thus, in such circumstances patients may find it difficult to experience peace. Therefore, in place of a state of peace and tranquility as included in the original English version of IPOS, the Hindi IPOS version referred to “carrying a feeling of peace” instead.

Two response categories namely “occasionally” and “overwhelmingly” were particularly challenging as they both have similar meanings in Hindi, so these two categories were difficult to differentiate. Similarly, “overwhelmingly” referred to the emotion of a person instead of rating the emotions on a degree or scale. This is similar to the difficulty of the response option “occasional” in IPOS validation in Turkish45 where this phrase carried a different meaning and needed to be changed.In an Indian health setting, patients were not used to rating their response on a five-point scale. The low education level of a large number of participants made it difficult for them to remember the five categories and then decide which option to choose. We therefore introduced a Visual Analog Scale adopted from the African Palliative Care Association African Palliative Outcome Scale (African APCA African POS)^42^.

In response to the first item of IPOS which asked patients their concerns in the past week, patients often shared at length their difficulties and problems which often exceeded the one-week timeframe. This suggests that patients need someone to listen to their concerns, thus a psycho social professional in the team may help patients to share their feelings. This also has practical implications, suggesting that for final administration of Hindi IPOS, professionals need to keep the patient focused on current concerns in the past week.

## Implications for use in practice

Despite the challenges experienced in translating individual phrases at the equivalence setting and forward translation phases, none of the professionals or patients found IPOS tiring or difficult to respond to. Patients saw it as an opportunity to share their deep-rooted concerns in a guided way. Similar experiences were reported by the teams working on IPOS translations in Italian^46^ and German^44^ suggesting the utility of IPOS in clinical settings across varied cultures.

This work has implications for patients across India and beyond. In north India, Hindi is the dominant language among people of all socio-economic groups, especially among less educated groups. In northern India, the capital city, Delhi, attracts patients from neighboring states as specialized services for public and private cancer care are available. As of the 2011 census, out of a total population of 1.2 billion in India, 52 million people reported Hindi as their mother tongue,^47^ 13 million reported Hindi as their second language, and another 2.4 million as their third language, suggesting a total of 57% of the Indian population speak Hindi.^48^ Across the world, Hindi was rated as the third most spoken language in 2019 with 615 million speakers worldwide.^49^ Hindi speaking populations across the world are not only in Asian countries, but also in US and European countries, and many others like Australia and New Zealand.^50^ IPOS Hindi will support these individuals to recognize their palliative care needs. The adaptation and validation of Hindi IPOS will be extended to patients living with advanced conditions including complexity and multiple morbidities.

## Limitations

The study was conducted through the community teams of a charitable palliative care organization that has its centers in the capital city of Delhi and neighboring regions. Hindi has slightly varied dialects across India, residents abroad may also have varying levels of proficiency in Hindi; this may prove to be a limitation of the tool. Future studies could include Hindi speaking respondents from more regions as well as non-oncology settings. The study was limited to community and oncology settings, with a lack of representation from hospice settings.

## Conclusion

The Hindi IPOS is relevant not only in Indian palliative care settings, but also for all the Hindi speaking patients settled elsewhere in the world who will now be able to utilize the tool to communicate their care priorities and needs. Further refinement may be needed for Hindi speakers living in different countries. The challenges faced in adapting a European measure into a different culture may provide an example of specific considerations that should be given when undertaking global adaptation and validation work. Our study shows that Hindi IPOS is simple to understand and relevant in capturing patient-centered outcomes in clinical practice and research. Psychometric validation of Hindi IPOS is underway.

## Supplemental Material

sj-pdf-1-pmj-10.1177_02692163221147076 – Supplemental material for Translation and cross-cultural adaptation of the Integrated Palliative Care Outcome Scale in Hindi: Toward capturing palliative needs and concerns in Hindi speaking patients Click here for additional data file. Supplemental material, sj-pdf-1-pmj-10.1177_02692163221147076 for Translation and cross-cultural adaptation of the Integrated Palliative Care Outcome Scale in Hindi: Toward capturing palliative needs and concerns in Hindi speaking patients by Tushti Bhardwaj, Rachel L Chambers, Harry Watson, Srividya, Irene J Higginson and Mevhibe B Hocaoglu in Palliative Medicine

sj-pdf-2-pmj-10.1177_02692163221147076 – Supplemental material for Translation and cross-cultural adaptation of the Integrated Palliative Care Outcome Scale in Hindi: Toward capturing palliative needs and concerns in Hindi speaking patients Click here for additional data file. Supplemental material, sj-pdf-2-pmj-10.1177_02692163221147076 for Translation and cross-cultural adaptation of the Integrated Palliative Care Outcome Scale in Hindi: Toward capturing palliative needs and concerns in Hindi speaking patients by Tushti Bhardwaj, Rachel L Chambers, Harry Watson, Srividya, Irene J Higginson and Mevhibe B Hocaoglu in Palliative Medicine

sj-pdf-3-pmj-10.1177_02692163221147076 – Supplemental material for Translation and cross-cultural adaptation of the Integrated Palliative Care Outcome Scale in Hindi: Toward capturing palliative needs and concerns in Hindi speaking patients Click here for additional data file. Supplemental material, sj-pdf-3-pmj-10.1177_02692163221147076 for Translation and cross-cultural adaptation of the Integrated Palliative Care Outcome Scale in Hindi: Toward capturing palliative needs and concerns in Hindi speaking patients by Tushti Bhardwaj, Rachel L Chambers, Harry Watson, Srividya, Irene J Higginson and Mevhibe B Hocaoglu in Palliative Medicine
