# Supplementary material for: Translation and cross-cultural adaptation of the Integrated Palliative Care Outcome Scale in Hindi: Toward capturing palliative needs and concerns in Hindi speaking patients
Source: Palliat Med. 2023 Jan 31;37(3):391–401. doi: 10.1177/02692163221147076 (PMC10021115; doi:10.1177/02692163221147076)
Supplement: sj-pdf-1-pmj-10.1177_02692163221147076 – Supplemental material for Translation and cross-cultural adaptation of the Integrated Palliative Care Outcome Scale in Hindi: Toward capturing palliative needs and concerns in Hindi speaking patients [file sj-pdf-1-pmj-10.1177_02692163221147076.pdf]

Appendix 1

Topic Guide to Interview Professionals

Integrated Palliative care Outcome Scale (IPOS) Pilot survey (Phase I)

Objective:

- To explore the cognitive processes used by respondents when reading, interpreting and responding to items on the IPOS questionnaire.

Introduction:

The respondents would be introduced about the study purpose, approximate time of engagement with interview, confidentiality, their right to stop any time and decline any questions

- I'm going to show you a questionnaire and I want you to read & answer the questions one at a time
- We will stop and talk about each question before moving onto the next
- Please try to 'think out loud' as you read and answer the questions (*DEMONSTRATE*)
- I will also ask you some more specific things about each question

I request you to recollect any of your patients you might have seen in the past week to answer these questions.

- In this study I am less interested in your answers to the questions, but *how you arrive* at the answers – what you think the question means, and the things you were thinking about when you chose your answer. I would also like to know any thoughts or views you might have about the questions

-- START INTERVIEW --

- *General:*

  - What were you thinking about when you answered that question?

- *Comprehension:* ***What does the respondent believe the question to be asking?***

  - What does the question mean to you, in your own words?

  - What does the word XXXXXX mean to you? (if certain words are thought to be problematic)

- How easy or difficult was it to understand this question?
- (If problem) How would you change this question?

- <u>*Retrieval*</u>*:* ***Could they recall the information required by the question? Was the time frame suitable?***

  - How well could you remember your experience when answering this question?
  - Was it easy or difficult to think about the past [week] when answering this question?
  - Would there be a different time period that would be easier to understand?

- <u>*Response:*</u> ***Is the respondent able to map their internally generated answer to a response option?***

  - How would you define the scoring keys of this question/ your interpretation of the scoring keys?
  - How did you choose your answer to this question?
  - Was it hard or easy to select an answer from the options given?
  - Did all options make sense for this question?

- <u>*Other* ***(to be asked at the end of all responses):***</u>

  - Is there anything else you would like to say about any of the questions? / Questionnaire as a whole?
  - Did you find any of the questions upsetting/ embarrassing / inappropriate?
  - Are there any topics/questions that you would leave out of this questionnaire?
  - Are there any topics/questions that you would add to this questionnaire?

--- THANKS ---

------------------------------------- COMPLETION OF DEMOGRAPHICS FORM ----------------------------------

DEMOGRAPHIC SHEET

Name (optional):

Institution:

Palliative care setting: Home care/ Hospice/In-house hospital/Day care/ Any other

Professional Profile: Doctor/Nurse/Psychologist/Social worker/ trained volunteer

Number of years of experience in Palliative care:

Professional qualification:

Age:

-- THANKS --
