# Supplementary material for: Translation and cross-cultural adaptation of the Integrated Palliative Care Outcome Scale in Hindi: Toward capturing palliative needs and concerns in Hindi speaking patients
Source: Palliat Med. 2023 Jan 31;37(3):391–401. doi: 10.1177/02692163221147076 (PMC10021115; doi:10.1177/02692163221147076)
Supplement: sj-pdf-2-pmj-10.1177_02692163221147076 – Supplemental material for Translation and cross-cultural adaptation of the Integrated Palliative Care Outcome Scale in Hindi: Toward capturing palliative needs and concerns in Hindi speaking patients [file sj-pdf-2-pmj-10.1177_02692163221147076.pdf]

Appendix 2

FGD Questions Guide

Item	Conceptual Recognition
**1.What have been your main problems/concerns over the past 3 days**	If we are interested to know about the problems faced by you, then:<br>1. Is the word "concern" confusing to understand?<br>2. Should we remove concern and keep only problem?<br>3. Is the time-frame of 3 days ok or you would suggest to increase it to 7 days?<br>4. What would you like to tell us if we ask you about your problems?
**2.Over the past 3 days, have you been affected by any of the symptoms: Pain/shortness of breath/weakness or lack of energy/ Nausea(feeling like you are going to be sick)/vomiting (being sick)/Poor appetite/ constipation/sore or dry mouth/Drowsiness/ Poor mobility**	1. Is the word "nausea" confusing to you?<br>2. Would Nausea and Vomiting give different meaning to you or they are the same?<br>3. Which word is more suitable "shortness of breath" or "breathing difficulty"?<br>4. For you Constipation is "hard stool" or inability to pass stool?<br>5. Does sore mouth or Dry mouth convey the same meaning to you or you suggest keeping these two as separate?<br>6. Do you think drowsiness or weakness carry same meaning?<br>7. Would you be more comfortable if given 3 options (**Not at all somewhat/ a little, very much)** to respond instead of 5 ("Not at all, slightly, moderately, severely, overwhelmingly"?<br>8. Are you able to respond poor appetite on the given response category?<br>9. Are slightly and overwhelmingly difficult to understand and respond?<br>10. Are you able to differentiate between slightly and moderately?
**3.Would you like to add any other symptoms to the above list**	Would you like to add loose motions/Diarrhoea to the above list?
**4. Over the past 3 days have you been feeling anxious or worried about your illness /treatment**	1. Should we ask you worries about **illness** and **treatment** separately or they are connected and can be asked in one question?<br>2. Would you like to drop the word "anxious" from this question and use only "worried"?

	3. Are you comfortable if we ask you "are you feeling tensed"?<br><br>4. Should we ask your feeling for the past 7 days or 3 days?<br><br>5. Would it be easy for you if we ask you to tell us your feeling for longer time period like one month 3 months?<br><br>6. Which response category is easier for you?<br>(i)Not at all, sometimes, most of the times<br><br>*(ii)* Occasionally, Sometimes, most of the times
**5.Have any of your family/friend being anxious or worried about you over the past 3 days**	1. Would the question be more simpler if we drop the word "anxious" and ask **"**Have any of your family/friend being worried about you" ?<br><br>2. While we are asking you if your family is worried about you, should we also ask you "are you worried about your family"? and<br><br>3. Should we also need to know "how well are you being taken care of by your family"<br><br>4 Would it be better if we ask this question for the past 7 days instead of 3 days?<br><br>5. Would you be more comfortable if we give you 3 categories to respond instead of 5 like:<br><br>Not at all, Sometimes, Most of the times/ always<br><br>6.which is a much better option: Most of the times or Always?
**6.Have you being feeling depressed over the past 3 days**	1 Are you more comfortable with the word "feeling Sad" or "being Depressed"?<br><br>2. Should we ask your feelings being sad/depressed during past 7 days or 3 days?<br><br>3. Which response category, you are more comfortable with:<br>1. Not at all, Sometimes, Always<br>2. Not at all, Sometimes, Most of the time<br>3. Not at all, occasionally, sometimes, most of the times, always
**7.Have you felt at peace over the past 3 days**	1. What do you understand if we ask you<br>(i) "Are you at peace"<br>(ii)"is your mind at rest"<br>(iii) are you satisfied with self?<br>2. Is this question easy to answer on<br>1. Not at all, Sometimes/occasionally, Most of the time

	**2.** Always, most of the time, sometimes, occasionally, not at all
**8.Over the past 3 days have you been able to share how you are you feeling with your family and friends as much as you wanted**	What do you understand if we ask you:<br>1. Are you able to share/ disclose your feelings with your family and friends?<br>2. Should we ask you this question for the past 7 days or longer time?<br>3. Would you be comfortable to respond this question on :<br>Not at all, sometimes and most of the times<br>4 Do you think that patients share their feelings "always" with their family?
**9. Over the past 3 days, have you had as much information as you wanted?**	1. If we want to know that you have as much information as you wanted, Would you be comfortable if we ask you:<br>• Do you have sufficient information about the illness or treatment<br>• Did you get information about the illness and treatment as you wanted?<br>2.Should we ask you this question for past 7 days or not put any time limit?<br>3. Are you comfortable responding to this item on ..<br>Not at all, Sometime, Most of the times
**10.Over the past 3 days, have any practical problems resulting from your illness being addressed (such as financial or personal)**	1. Does the question "Have your personal and financial problems resulting from illness being addressed" clear to you?<br>2. Should we ask your personal and financial problems separately in two questions?<br>***3.*** Does the personal problem mean problems in your family relations?<br>***4.*** Should this question be asked for past 7 days or longer time like a month?<br>***5.*** Which response category is more comfortable?<br>(i) Problems addressed, Problems not addressed<br>(ii) Problems addressed, some problems addressed, problems not addressed
**How did you complete this questionnaire**	Is this easy or hard for you to understand?
