# Supplementary material for: Translation and cross-cultural adaptation of the Integrated Palliative Care Outcome Scale in Hindi: Toward capturing palliative needs and concerns in Hindi speaking patients
Source: Palliat Med. 2023 Jan 31;37(3):391–401. doi: 10.1177/02692163221147076 (PMC10021115; doi:10.1177/02692163221147076)

स्टाफ के प्रयोग के लिए
मरीज़ का नंबर

आई.पी.ओ.एस.मरीज की प्रश्नावली

www.pos-pal.org

नाम: ....
दिनांक:

कृपया एक खाने में एक अक्षर या संख्या साफ-साफ लिखें। आपके जवाब हमें आपकी व औरों की देखभाल बेहतर करने में मदद करेंगे।

धन्यवाद।

प्रश्न 1. पिछले सप्ताह में आपकी मुख्य परेशानियां क्या रही हैं?

1. ....
2. ....
3. ....

प्रश्न 2. नीचे कुछ परेशानियों की सूची दी गई है, जो हो सकता है आपने पिछले सप्ताह में अनुभव की हों। प्रत्येक परेशानी के सामने दिए गए बॉक्स में (√) का निशान लगाएं, जो हमें बताएगा कि इसने आपको पिछले सप्ताह में कैसे प्रभावित किया है?
(टिप्पणी: मरीज़ को उत्तर देने में आसानी के लिए अंतिम पृष्ठ पर दिए थर्मामीटर का प्रयोग करें)

परेशानियां	बिल्कुल नहीं 0	बहुत कम 1	थोड़ा बहुत 2	ज्यादा 3	बहुत ज्यादा 4
दर्द					
सांस लेने में कठिनाई					
कमजोरी या ताकत की कमी					
उल्टी जैसा लगना					
उल्टी आना					
कम भूख लगना					
कब्ज/ पेट का अच्छे से साफ़ न होना					
मुंह में छाले या मुँह का सूखना					
अधिकतर समय नींद आना					
चलने-फ़िरने में दिक्कत					

क्या कोई और परेशानी है, जो आपने पिछले सप्ताह में महसूस की हो? कृप्या नीचे दिए गए बॉक्स में निशान (√) लगा कर बताएं ।

परेशानी	बिल्कुल नहीं 0	बहुत कम 1	थोड़ा बहुत 2	ज्यादा 3	बहुत ज्यादा 4
1.<br>2.<br>3.					

पिछले सप्ताह में:

(टिप्पणी: मरीज़ को उत्तर देने में आसानी के लिए अंतिम पृष्ठ पर दिए थर्मामीटर का प्रयोग करें)	बिल्कुल नहीं<br>0	बहुत ही कम बार<br>1	कभी कभी<br>2	अधिकतर<br>3	हमेशा<br>4
प्र.3. क्या आप बीमारी और इलाज को लेकर मानसिक तनाव में या चिंतित रहें हैं?					
प्र.4. क्या आपके परिवार और मित्रों में कोई आपको लेकर घबराहट में या चिंतित रहें हैं?					
प्र.5.क्या आपने उदासीनता महसूस की हैं?<br>(उदासीनता: निराशा, अलग-थलग रहना, बहुत दु:खी रहना, अक्सर रोना)					

पिछले सप्ताह में:

(टिप्पणी: मरीज़ को उत्तर देने में आसानी के लिए अंतिम पृष्ठ पर दिए थर्मामीटर का प्रयोग करें)	हमेशा<br>0	अधिकतर<br>1	कभी कभी<br>2	बहुत ही कम बार<br>3	बिल्कुल नहीं<br>4
प्र.6. क्या आपने मन की शांति महसूस की है?					
प्र.7. क्या आप अपनी भावनाएं परिवार एवं मित्रों को बता पाएं?					
प्र.8. क्या आपको अपनी बीमारी, इलाज तथा किसी अन्य विषय की पर्याप्त जानकारी मिली ?					

	समस्याओं का पूरी तरह से समाधान/ कोई समस्या ही नहीं<br>(0)	अधिकतर समस्याओं का समाधान<br>(1)	कुछ समस्याओं का समाधान<br>(2)	ना के बराबर समाधान<br>(3)	किसी भी समस्याओं का समाधान नहीं<br>(4)
प्र.9 क्या आपकी बीमारी और इलाज से जुड़ी समस्याओं का समाधान हुआ (जैसे की खर्चे की या निजी)					

	अपने आप	दोस्त या रिश्तेदार की मदद से	स्वास्थ्य कर्मचारी की मदद से
प्र.10 इस प्रश्नावली को आपने कैसे पूरा किया?			

यदि आप इस प्रश्नावली में उठाए गए मुद्दों में से किसी को लेकर चिंतित हैं, तो कृपया अपने डॉक्टर या नर्स को बताएं।

ज़वाब देने का थर्मामीटर

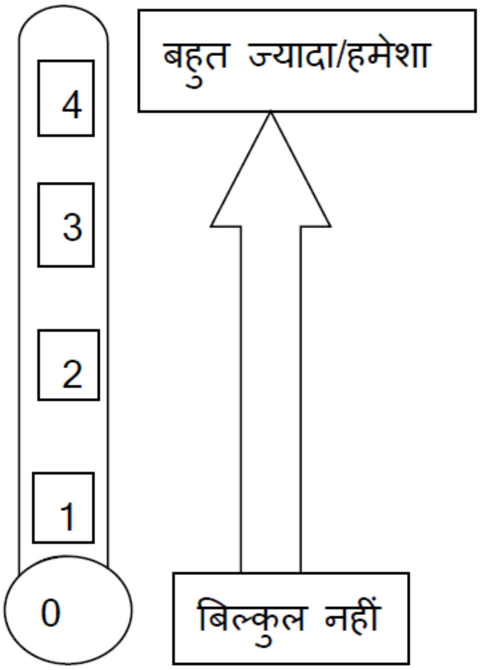

Supplement: sj-pdf-3-pmj-10.1177_02692163221147076 – Supplemental material for Translation and cross-cultural adaptation of the Integrated Palliative Care Outcome Scale in Hindi: Toward capturing palliative needs and concerns in Hindi speaking patients [file sj-pdf-3-pmj-10.1177_02692163221147076.pdf]
